# Association of *PARP1*-specific polymorphisms and haplotypes with non-small cell lung cancer subtypes

**DOI:** 10.1371/journal.pone.0243509

**Published:** 2020-12-07

**Authors:** Jing Jin, Heather Robeson, Pebbles Fagan, Mohammed S. Orloff

**Affiliations:** 1 Department of Epidemiology, University of Arkansas for Medical Sciences, Little Rock, Arkansas, United States of America; 2 Department of Health Behavior and Health Education, University of Arkansas for Medical Sciences, Little Rock, Arkansas, United States of America; 3 Winthrop P. Rockefeller Cancer Institute, University of Arkansas for Medical Sciences, Little Rock, Arkansas, United States of America; 4 Center for the Studies of Tobacco, Fay W. Boozman College of Public Health, University of Arkansas for Medical Sciences, Little Rock, Arkansas, United States of America; University of North Carolina at Chapel Hill, UNITED STATES

## Abstract

**Objective:**

The carcinogenesis role of *PARP1* in lung cancer is still not clear. Analysis at allelic levels cannot fully explain the function of *PARP1* on lung cancer. Our study aims to further explore the relation between *PARP1* haplotypes and lung cancer.

**Materials and methods:**

DNA and RNA were extracted from non-small cell lung cancer (NSCLC) tumor and adjacent normal fresh frozen tissue. Five *PARP1*-SNPs were genotyped and *PARP1*-specific SNPs were imputed using IMPUTE and SHAPEIT software. The SNPs were subjected to allelic, haplotype and SNP-SNP interaction analyses. Correlation between SNPs and mRNA/protein expressions were performed.

**Results:**

SNP imputation inferred the ungenotyped SNPs and increased the power for association analysis. Tumor tissue samples are more likely to carry rs1805414 (OR = 1.85; 95% CI: 1.12–3.06; P-value: 0.017) and rs1805404 (OR = 2.74; 95%CI 1.19–6.32; P-value: 0.015) compared to normal tissues. Our study is the first study to show that haplotypes comprising of 5 SNPs on *PARP1* (rs1136410, rs3219073, rs1805414, rs1805404, rs1805415) is able to differentiate the NSCLC tumor from normal tissues. Interaction between rs3219073, rs1805415, and rs1805414 were significantly associated with the NSCLC tumor with OR ranging from 3.61–6.75; 95%CI from 1.82 to 19.9; P-value<0.001.

**Conclusion:**

*PARP1* haplotypes may serve as a better predictor in lung cancer development and prognosis compared to single alleles.

## 1. Introduction

Lung cancer patients have the highest mortality rate among all cancer patients [1]. In 2020, there would be around 228,820 new cases of lung cancer, and 135,720 deaths from lung cancer in the United States [2]. Non-small cell lung cancer (NSCLC) is the most common subtype (80%-85%), which can be further classified into adenocarcinoma (AC), squamous cell carcinoma (SCC), and large cell carcinoma [3]. The five-year survival rate of lung cancer is around 56% for early-stage cases [4]. However, only 16% of lung cancer patients can be detected at an early stage [4]. The currently recommended lung cancer screening for high-risk population is a low-dose computed tomography CT scan (LDCT) [5], but the test shows a high rate of noncalcified nodules and high cost of follow-up tests [6]. While multiple known environmental factors are associated with lung cancer [7], genetic mutations have also been shown to have important roles. Genetic variations in genes like the *TP53*, *EGFR*, and *KRAS* have been identified and are currently used as biomarkers in clinical practice, but are only effective for selected lung cancer patients [8–12]. Therefore, unexplored gene mutations could help explain the poor prognosis of lung cancer [13], and further the treatment effectiveness for specific subtypes of lung cancer [14]. The heterogeneity and complexity of the lung cancer disease require more efforts in investigating the potential role of novel genes that are associated with lung cancer development.

One such gene is Poly (ADP-ribose) polymerase 1 (*PARP1*), which is an enzyme that has an important role in transcriptional control, DNA repair machinery, differentiation, proliferation, and cell death [15, 16]. The role of *PARP1* in lung cancer is yet to be fully understood. Some studies have implicated PARP inhibitor as a therapeutic target in cancer treatment [17, 18]. The combinatorial treatment using *PARP1* inhibitors and DNA-interacting chemotherapeutic agents was shown to increase the radio sensitivity of the lung tumor [19] and pharmacological *PARP1* inhibitor may increase the chemo-sensitivity of NSCLC through *PARP1*-dependent DNA repair [20]. Studies have also shown allelic heterogeneity in PARP1 in specific populations associated with lung cancer. For example, rs3219073 C>G polymorphism in *PARP1* is associated with lung cancer susceptibility, and the carriers of G alleles may have reduced risk of lung AC [21]. Along, these same lines, haplotypes derived from three polymorphisms derived from different locations of the *PARP1* in an Asian population was not associated with lung cancer [22] and the study failed to test association with the lung cancer subtypes.

Taking all this together in conjunction with a recent finding suggesting that the inhibition of *PARP1* can help curb inflammation-associated NSCLC development, specifically lung AC [23], it is likely that *PARP1* may have a role in lung carcinogenesis and warrants rigorous characterization and understanding the role of *PARP1* in promoting NSCLC subtype development. Therefore, our goal was to genotype *PARP1* SNPs in paired NSCLC tumor and normal tissues in a population of European ancestry, construct haplotypes and test for association with NSCLC subtypes and *PARP1* expression at the transcript and protein levels.

## 2. Materials and methods

### 2.1 Subjects recruitment and tissue procurement

The study included 83 patients who had NSCLC as primary cancer, aged 35–85 years old, and received tumor resection surgery in the University of Arkansas for Medical Sciences (UAMS). Trained pathologists performed the NSCLC histological classification.

We included 124 NSCLC tumor and adjacent normal fresh frozen tissues and 21 unpaired fresh frozen lung tissues in the study. We obtained all samples from the Tissue Procurement Facility at the University of Arkansas for Medical Sciences (UAMS). Surgical lung cancer tissue samples were collected during tumor resection surgery and snap-frozen in liquid nitrogen immediately to decrease hypoxic phenomena on gene expression. Matched adjacent normal tissues were also obtained around 2–5 cm away from the tumor margin. All specimens were stored at –80°C. All procedures were under aseptic conditions and followed the standardized protocol. This is a retrospective study using existing data and tissue and there were no direct contact with the study participants. This study has obtained IRB (protocol reference number 202880) approval from the University of Arkansas for Medical Sciences, Institutional Review Board.

### 2.2 Tissue homogenization

A 10–20 μm section was carefully excised from each of the patient-derived frozen tissues. The samples were transferred to a ceramic mortar, and liquid nitrogen was added immediately. The tissues were crushed gently to a fine powder. The powdered samples were immediately transferred into a 2 ml microcentrifuge tube and homogenized in 600 μl RLT buffer (Qiagen, Valencia, CA, USA) with β-mercaptoethanol three times for 30secs at medium speed using a D1000 MidSci Hand-held Tissue Homogenizer (MidSci, St. Louis, MO, USA).

### 2.3 RNA and DNA extraction

DNA was extracted using the ALLprep DNA/RNA/protein Mini Kit [24]. Biological samples were lysed and homogenized first in highly denaturing guanidine isothiocyanate–containing buffer to inactivate the DNases, RNases, and proteases. The lysate was passed through the spin column, which contains a high-salt buffer to allow the effective and efficient binding of genomic DNA. The DNA was finally eluted after washing and purification for both lung tumor tissues and adjacent normal tissues using the Qiagen All Prep DNA/RNA/Protein Mini Kit according to the manufacturer's instructions [24].

RNA was extracted using the Qiagen’s AllPrep DNA/RNA/Protein Mini Kit [24] from homogenized tissue samples according to manufacturer’s protocol. After eluting RNA using 40μL of RNAse-free water, samples were stored at -80°C for later Complementary DNA (cDNA) synthesis and qRT-PCR analysis.

### 2.4 Reverse transcription

cDNA was synthesized using the High Capacity RNA- to -cDNA Kit (Applied Biosystems, Carlsbad, CA, USA) according to manufacturer’s protocol [25]. Thermocycler conditions were as follows: 37°C for 60 min, 95°C for 5 min, and then held for 4°C. Varying cDNA concentrations across samples were then diluted (1:10) in nuclease-free water.

### 2.5 Real-time polymerase chain reaction (qPCR)

cDNA from lung carcinoma tissue samples of various subtypes were used to perform real-time PCR [25]. Amplification of target gene *PARP1* in carcinoma and adjacent carcinoma tissue samples was assessed. *HPRT*, a control gene that has been identified as being stable for use with lung samples, was used to normalize all values to a point and ensure that the effect of varying DNA content is minimized. Primers were designed specifically targeting the exonic regions of the *PARP1* gene using the USCS (build 19) *in-silico* PCR and Integrated DNA Technologies assay design tool. *PARP1* forward primer sequence used was “CCTCCGCTCCTGAACAATG” and the *PARP1* reverse sequence used was “TGAGCTTCTCATAGTTGACATCG”.

### 2.6 *PARP1* mRNA expression validation analysis from publicly available dataset

We compared *PARP1* mRNA expression levels with publicly available microarray dataset. Specific inclusion, exclusion and appropriate quality checks (S1 Table in S2 File) were applied to obtain Gene Expression Omnibus (GEO) datasets from ONCOMINE (www.oncomine.org). ONCOMINE is a web-based data-mining platform comprising of cancer microarray data. We selected Hou et al. lung dataset (GSE 19188) [26] as the validation set of our study based on sample size, histological subtyping of lung tissue samples, and data quality.

### 2.7 Enzyme-linked immunosorbent assay (ELISA)

Total proteins isolated with the AllPrep DNA/RNA/Protein Mini Kit were quantitated using the BCA Protein Assay Kit (Thermo Scientific Pierce, Grand Island, NY, USA). The Human *PARP1* DuoSet ELISA kit and the DuoSet Ancillary Reagent kit from R&D Systems (Minneapolis, MN) were used to perform the ELISA assays as per manufacturer’s instructions [27]. Extracted protein samples were prepared at a 1:20 or 1:50 dilution for the assay. We used *PARP-1* antibody (F-2), catalog number sc-8007 from Santa Cruz Biotechnology. The epitope is for amino acids position 764–1014 at the C-terminus of *PARP1* human origin. Recombinant human *PARP1* standard provided with the ELISA kit was used to create a seven-point standard curve. A reagent blank was included in the ELISA assays, consisting of the reagent diluent provided in the ancillary reagent kit with no protein sample added. Each standard, sample and reagent blank were run in duplicate when performing the ELISA. Results of the ELISA assays were read using a SpectraMax M5 microplate reader (Molecular Devices, LLC, San Jose, California) at a wavelength of 450 nm, and analyzed using SofMaxPro software v. 6.5.1 (Molecular Devices, LLC, San Jose, California) provided with the microplate reader.

### 2.8 SNP genotyping and SNP imputation

For SNP genotyping, 5 SNPs in *PARP1* were selected based on minor allele frequencies, functionality, location in functional domains, transcriptional factor (TF) binding sites, enhancer or promoter activities, etc. TaqMan SNP Genotyping Assays (Applied Biosystems, Foster City, CA) was used and standard quality control procedures were followed [28]. Data with low call-rate (<95%) were excluded to improve the stability of test statistics and performed genotyping replication. The allele frequencies were also compared with race-specific reference allele frequencies on the University of California, Santa Cruz (UCSC) genome browser.

We performed SNP imputation to infer the alleles of ungenotyped variants and to fine map putative functional domains. The reference panel is 1000 Genomes phase 3 collection. We used IMPUTE version 2 software [29] to impute SNPs from a reference panel to study panel based on pre-phased haplotypes. We used SHAPEIT [30] for pre-phasing analysis. We excluded genotyped SNPs with low minor allele frequency (MAF<2%), high individual missingness rate (>80%), and high genotyping missingness rate (>80%). We targeted the interval of genotyped *PARP1* SNPs and 250 kilobase-pair flanking the interval.

### 2.9 Statistical analysis

#### 2.9.1 Genotypic and allelic association analysis

The PLINK toolset version 2.05 [31] for the whole-genome association was used to perform association analysis. We used SNPs with minor allele frequency (MAF) above 2%. We excluded SNPs with high individual missingness rate (>5%), high genotyping missingness rate (>5%), and Hardy-Weinberg equilibrium with P value ≤0.001.

#### 2.9.2 SNP-SNP interaction using multifactor dimensionality reduction (MDR) analysis

To identify combinations of multilocus genotypes that are associated with high risk of lung cancer and combinations that are associated with low risk of lung cancer, we conducted MDR analysis [32] to detect and characterize high-order SNP-SNP interactions. MDR method can address the limitation of traditional logistic regression regarding the curse and dimensionality. Both genotyped and imputed SNPs were included in the analysis.

#### 2.9.3 Linkage disequilibrium (LD) and haplotype analysis

**Fig 1:**
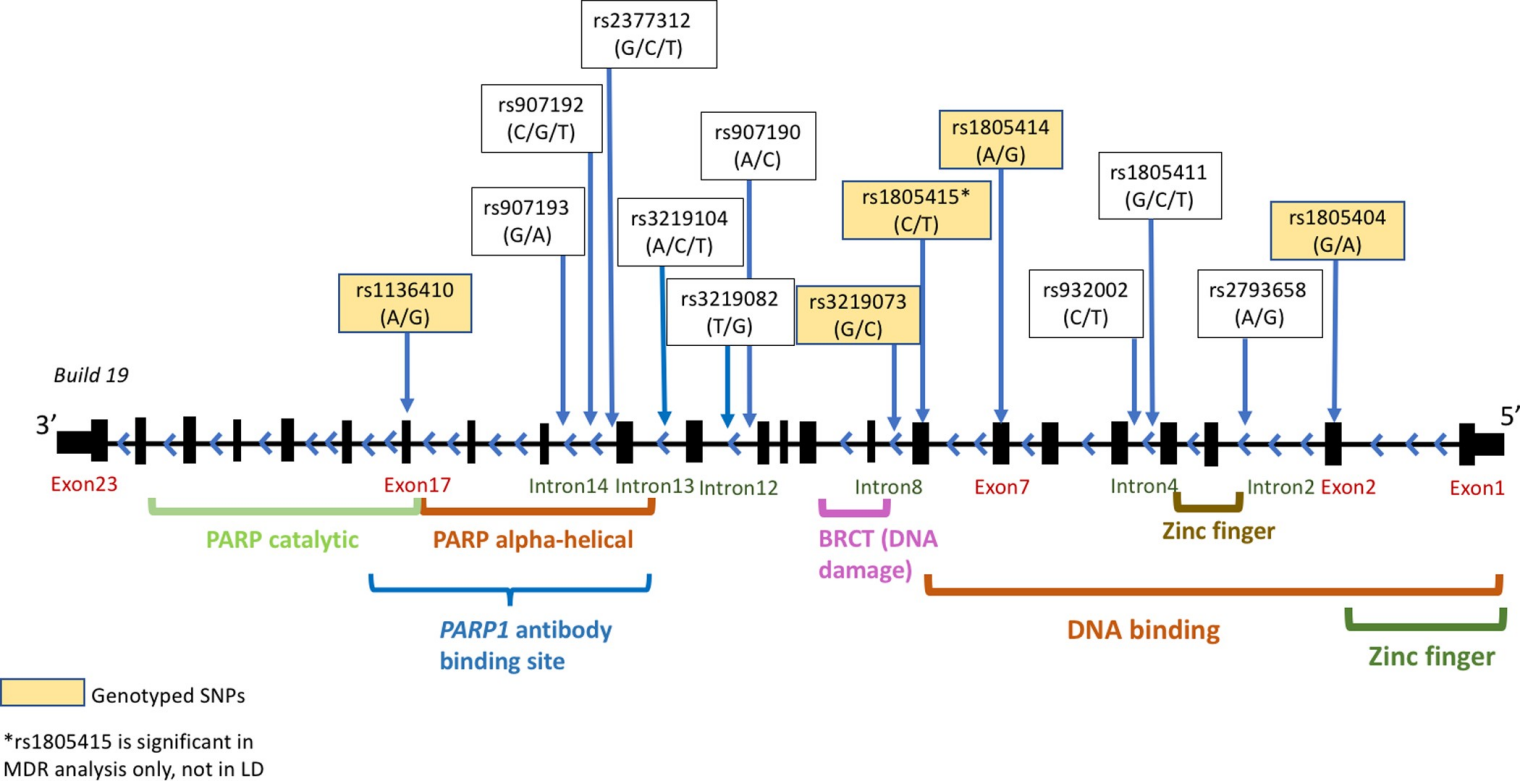
Depiction of the *PARP1* gene showing the SNPs analyzed and how they map onto key *PARP1* domains. The figure shows the locations of genotyped and imputed SNPs that passed quality check spanning the 34656bp *PARP1* gene. The genotyped SNPs are in yellow boxes and imputed SNPs are in white boxes. The labels below the gene show the locations of important functional domains. In addition, the figure shows the antibody binding site that contains both *PARP* catalytic and *PARP* alpha-helical domains. The *PARP1* antibody binding site guided the ELISA experiment. The rs1805415 is a tag SNP within an LD block was significant in the MDR analysis. All SNPs (except rs1805415*) in LD blocks were from results of all subtypes of NSCLC.

We performed LD analysis to identify the effect of multiple SNPs on *PARP1*, which measures the allelic association by taking the difference between the actual haplotype frequency and the equilibrium value. For a more refined LD block determination and finer haplotype reconstruction, we needed more SNPs, hence both genotyped and imputed SNPs were used. We evaluated standardized pairwise linkage disequilibrium LD (D’) for SNPs between pairs of alleles at different loci, and the results were visualized in HAPLOVIEW software (version 4.2) using PLINK software (version 2.05) [31]. SNPs within a block of LD that defines *PARP1* based on linkage disequilibrium (Fig 1) were used to infer phase and reconstruct haplotypes using the PHASE software (version 2.1.1) [33, 34]. The haplotypes generated by PHASE software were analyzed based on molecular diversity indices [33, 34]. We computed a minimum spanning tree among haplotypes based on pairwise differences [35] in Arlequin software (version 3.5) [36]. Individual specific haplotypes were then generated, and the haplotype frequencies between tumor and normal samples or between subtypes of lung cancer samples were compared. To obtain reliable results, we performed 5000 iterations and 100 burn-in times in the PHASE software to reliably generate haplotype trees. We performed 100 permutation tests for significant differences in haplotype frequencies in case and control groups in PHASE, which increases the power to test unique haplotype in the sample [37].

#### 2.9.4 *PARP1* mRNA expression analysis and validation using publicly available microarray dataset (GSE19188)

A comparison of *PARP1* expression between AC or SCC tissues and adjacent normal tissues was performed using Prism 8 (GraphPad Software, Inc., La Jolla, CA, USA). Statistical tests included paired (N = 62 pairs) t-test, unpaired t-test (N = 145: 124 paired and 21 unpaired samples) and one-way ANOVA test.

A publicly available dataset (GSE19188) (N = 156) was used to validate the mRNA expression results. *PARP1* expression microarray data were downloaded and imported to GraphPad Prism 8 (GraphPad Software, Inc., La Jolla, CA, USA). The normalized values for normal lung tissues were compared to normalized values for AC tissues and for SCC tissues using a t-test. Data are presented as mean ±SEM.

#### 2.9.5 Protein expression analysis

*PARP1* protein expression data derived from ELISA using a subset of the paired samples [N = 58] were imported into GraphPad Prism 8. The normalized values for paired normal lung tissues were compared to normalized values for AC tissues and for SCC tissues using a paired and unpaired t-test. Data are presented as mean ±SEM. The purpose of this data was to functionally validate significant genotypes.

## 3. Results

### 3.1 Subjects and tissue samples information

Total of 83 subjects who were diagnosed as NSCLC were included in this study, among which 62 of them had paired tumor and normal tissues collected. The total number of samples is 145 (S2 Table in S2 File). The sample information is shown in S2 Table in S2 File.

### 3.2 SNP imputation

In the effort to infer ungenotyped SNP information, the imputation of *PARP1*-specific SNP was performed. SNP imputation uses a set of reference haplotypes to estimate the unobserved variants that are not directly genotyped as described by Li et al., 2009 [38]. The power of association analysis can be increased through imputing SNPs. Five SNPs were genotyped and included in the SNP imputation process (S3 Table in S2 File). In the SNP imputation process, we excluded low-quality imputed SNPs based on individual missingness rate (>5%), SNP missingness rate (>5%). We included imputed SNPs with low minor allele frequency. High LD, haplotype blocks, and 71.3% concordance together provided reliability in the accuracy of the imputation. Variability of the imputation could have been caused by presence of the rare alleles in the untyped or imputed SNPs [39, 40]. SNP density in the region which could have slightly lowered the accuracy score [41]. As a result, there were 1013 SNPs in total after imputation.

### 3.3 Genotypic and allelic association analysis after imputation

Genotypic and allelic association analyses were conducted. SNPs rs1805414 [G] and rs1805404 [A] were significantly associated, and rs1136410 [G] was associated with a p-value of 0.062 with lung tumor when compared to normal tissue samples (Table 1). The alleles of nine imputed and two genotyped (rs1805414 [G] and rs1805404 [A]) SNPs were significantly associated with SCC compared to normal tissues samples (Table 1). There were no significant SNPs identified when AC as compared to SCC (Table 1).

**Table 1:** Allelic and genotypic association after imputation.

Allelic association					Genotypic association	
SNPs	Location	Minor allele /MAF	OR (95%CI)	P value	Model	P value
	(Build Hg19)					
**Tumor versus Normal tissues**						
**rs1136410**	226555302	G/0.095	2.218 (0.944, 5.212)	0.062	Trend	0.090
**rs1805414**	226573364	G/0.330	1.848 (1.115, 3.064)	0.017	Geno	0.031
					Trend	0.024
					Dominant	0.009
**rs1805404**	226589958	A/0.109	2.739 (1.187, 6.318)	0.015	Trend	0.023
**SCC versus Normal tissues**						
rs752307	226551529	C/0.218	3.448 (1.081, 11)	0.028	Trend	0.065
rs907193	226561188	G/0.269	2.326 (1.209, 4.472)	0.010	Trend	0.014
rs907192	226561225	C/0.269	2.326 (1.209, 4.472)	0.010	Trend	0.014
rs2377312	226561761	G/0.269	2.326 (1.209, 4.472)	0.010	Trend	0.014
rs3219104	226562621	A/0.077	3.448 (1.081, 11)	0.028	Trend	0.065
rs3219082	226566401	G/0.079	3.875 (1.244, 12.07)	0.013	Trend	0.035
rs907190	226566726	A/0.269	2.326 (1.209, 4.472)	0.010	Trend	0.014
**rs1805414**	226573364	G/0.330	2.016 (1.079, 3.765)	0.027	Trend	0.043
					Dominant	0.029
rs932002	226577306	T/0.079	3.814 (1.224, 11.88)	0.014	Trend	0.038
rs1805411	226578047	T/0.077	2.897 (0.961, 8.73)	0.050	Trend	0.096
**rs1805404**	226589958	A/0.109	2.716 (1.019, 7.238)	0.039	Trend	0.067
**AC versus SCC (or normal) tissues (not significant)**						

### 3.4 SNP-SNP interaction analysis

The MDR analysis was performed to identify SNP-SNP interactions in the *PARP1* gene that could confer risk to NSCLC types. MDR reduced the dimensionality of large amount of SNP data and improved the ability to identify SNP-SNP combinations that are associated with lung cancer risk. MDR categorized the genotypes into low-risk and high-risk groups to reduce the dimensionality. The traditional logistic regression model may introduce increased type I errors and large standard errors due to the increase of a large number of possible interactions. We used MDR to characterize epistasis models. The two- and three-way SNP-SNP interaction models for the common SNPs in *PARP1* by comparison group are listed in Table 2. For the comparisons, tumor versus normal, SCC versus normal and AC versus normal, the two-way and three-way interactions had the same SNPs that were significant i.e., rs3219073, rs1805415, and rs1805414 (Table 2). For the comparisons, AC versus SCC, the two-way and three-way interactions had the SNPs that were significant, including rs1136410, rs3219073, and rs1805415 (Table 2).

**Table 2:** Display of significant *PARP1* SNP-SNP interactions.

**Tumor vs Normal**								
**Rank**	**Model**			**Training Bal. Acc. (%)**	**Testing Bal. Acc. (%)**	***Cross-Validation Consistency***	**Odds ratio (95%CI)**	**P-Value**
**One-Way**	rs1805414			63.18%	63.18%	10/10	2.949 (1.507, 5.771)	0.001
**Two-Way**	rs1805415	rs1805414		64.12%	59.35%	6/10	3.134 (1.597, 6.149)	0.0008
**Three-Way**	rs3219073	rs1805415	rs1805414	67.67%	59.55%	7/10	3.611 (1.823, 7.153)	0.0002
**SCC vs Normal**								
**One-Way**	rs1805414			64.59%	64.59%	10/10	3.344 (1.368, 8.174)	0.007
**Two-Way**	rs1805415	rs1805414		66.91%	65.3%	10/10	4.137 (1.658, 10.325)	0.002
**Three-way**	rs3219073	rs1805415	rs1805414	68.64%	55.62%	7/10	4.685 (1.869, 11.748)	0.0006
**AC vs Normal**								
**One-Way**	rs1805414			61.11%	57.86%	9/10	2.461 (1.002, 6.043)	0.047
**Two-Way**	rs3219073	rs1805414		65.12%	59.64%	10/10	5.083 (1.824, 14.164)	0.001
**Three-way**	rs3219073	rs1805415	rs1805414	66.59%	60%	9/10	6.75 (2.288, 19.901)	0.0002
**AC vs SCC**								
**One-Way**	rs1805414			55.3%	32.37%	4/10	1.359 (0.467, 3.957)	0.573
**Two-Way**	rs3219073	rs1805414		60.95%	47.41%	9/10	2.648 (0.838, 8.368)	0.092
**Three-way**	rs1136410	rs3219073	rs1805415	62.99%	44.351%	6/10	3.020 (0.959, 0.507)	0.055

### 3.5 Linkage disequilibrium and haplotype analysis after imputation

#### 3.5.1 The D’ linkage disequilibrium (LD) patterns of the *PARP1* gene after imputation

**Fig 2:**
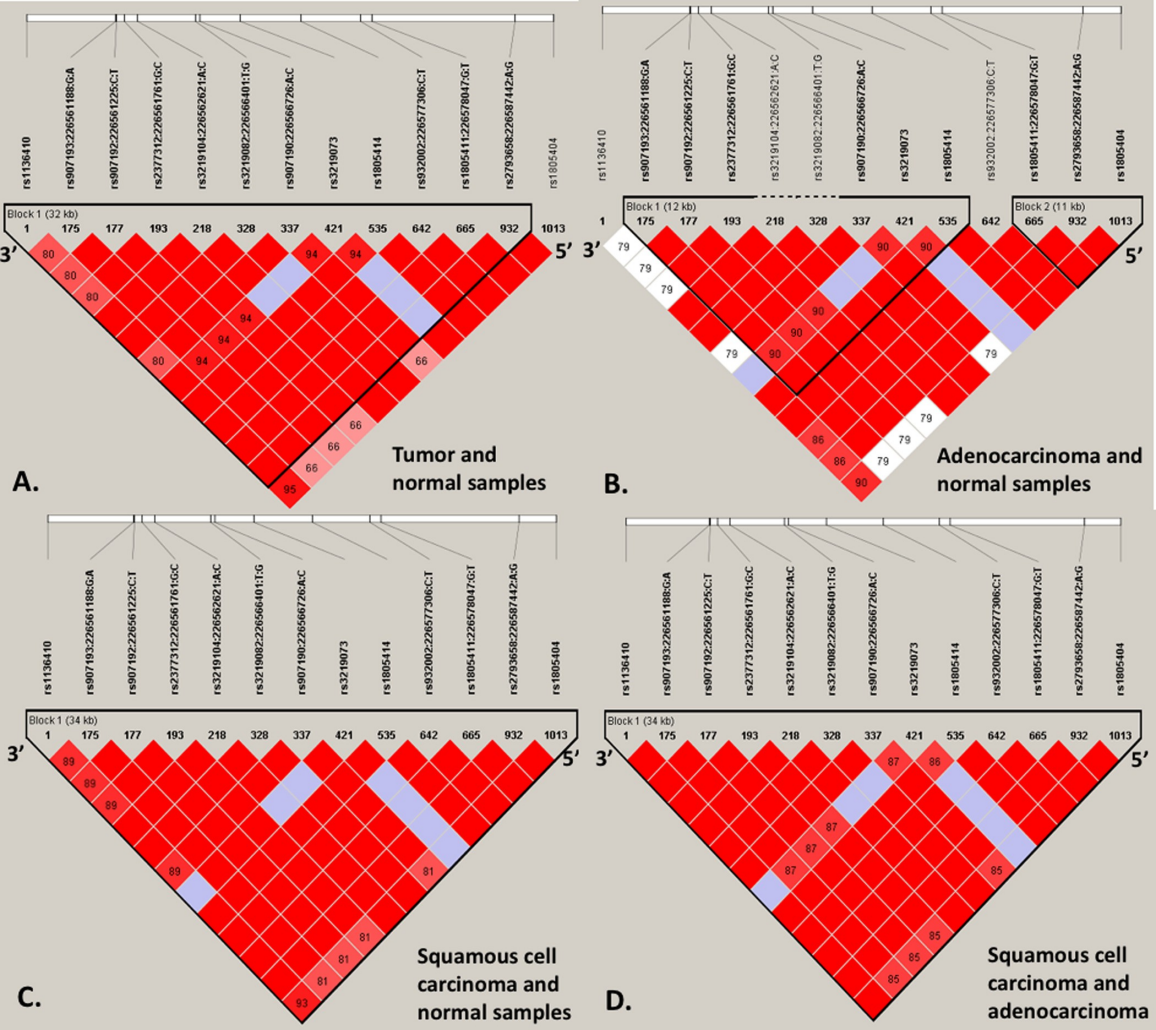
Pairwise LD analysis of lung cancer samples and by lung cancer subtypes. A. The figure displays LD plot of tumor and normal samples defined by 4 genotyped and 8 imputed SNPs covering a 32Kb block size. SNPs rs1136410, rs3219073, rs1805414, and rs1805404 were genotyped in the lab. The red coloration implies a relatively strong LD with a D’> 0.8. A block is defined by the black triangle line and was constructed if 95% of informative pair-wise comparisons were in strong LD. The numbers in the plot represents the crossover percentage to the next block. SNP rs1805404 is a tag SNP since it was highly correlated with rs1805414 before imputation, S1 Fig in S1 File (i.e. for imputation tag SNPs are normally used). B. The LD plot of AC and normal samples is defined by 2 genotyped and 6 imputed SNPs covering a 12Kbp block size. The block included genotyped SNPs rs3219073 and rs1805414. We defined a relatively strong LD pattern with a pairwise D’> 0.8. A block was created if 95% of informative comparisons were in strong LD. Three SNPs are highly correlated or in tight LD after imputation with a block size of 11kb (on the right). Note that rs932002 was included in the haplotype analysis since it is a tag SNP. C. The LD plot of SCC and normal samples is defined by 4 genotyped and 9 imputed SNPs covering a 34Kbp block size. The LD block included genotyped SNPs rs1136410, rs3219073, rs1805414, and rs1805404. D. The LD plot of SCC and AC samples is defined by 4 genotyped and 9 imputed SNPs covering a 34Kbp block size. The LD block contains genotyped SNPs rs1136410, rs3219073, rs1805414, and rs1805404.

A relatively strong LD pattern was defined as having a pairwise D’> 0.8. For all samples after imputation, 12 SNPs (i.e. rs1136410, rs907193, rs907192, rs2377312, rs3219104, rs3219082, rs907190, rs3219073, rs1805414, rs932002, rs1805411, and rs2793658) were highly correlated with each other and together defined a block [32 Kilobase-pair] shown in Fig 2A. The MAFs of these respective SNPs are shown in S3 Table in S2 File. Rs1805404 was not among the SNPs that defined the block but is a tag SNP for rs1805414 based on the tight LD between them before imputation (S1 Fig in S1 File).

#### 3.5.2 Comparison of the D’ linkage disequilibrium (LD) patterns of the *PARP1* gene between lung tumor tissue samples and lung normal tissue samples

To assess the differences in allele frequencies that drive the LD patterns in the tumor and normal tissues, we made separate D’ LD plots of the *PARP1* gene for the lung tumor tissue samples and lung normal tissue samples to examine the differences in the LD pattern of *PARP1* SNPs between lung tumor (block size: 12 kilobase-pair) and normal tissues (block size: 32 kilobase-pair) (S3 and S4 Figs in S1 File). Pairwise LD analysis showed differences in the sizes and patterns of LD block between tumor and normal tissue samples (S3 and S4 Figs in S1 File). Pairwise LD analysis showed differences in the patterns of LD block between AC (block size: 5 kilobase-pair, 10 kilobase-pair) and SCC tissue samples (block size: 34 kilobase-pair) (S5 and S6 Figs in S1 File). We also generated LD plots after combining the samples based on comparison groups (Fig 2). The SNPs for haplotype analysis were selected based on the LD blocks of different comparison groups: tumor and normal (Fig 2A, 12 SNPs), AC and normal (Fig 2B, 12 SNPs including rs932002), SCC and normal (Fig 2C, 13 SNPs), and AC and SCC (Fig 2D, 13 SNPs).

#### 3.5.3 Haplotype analysis

**Fig 3:**
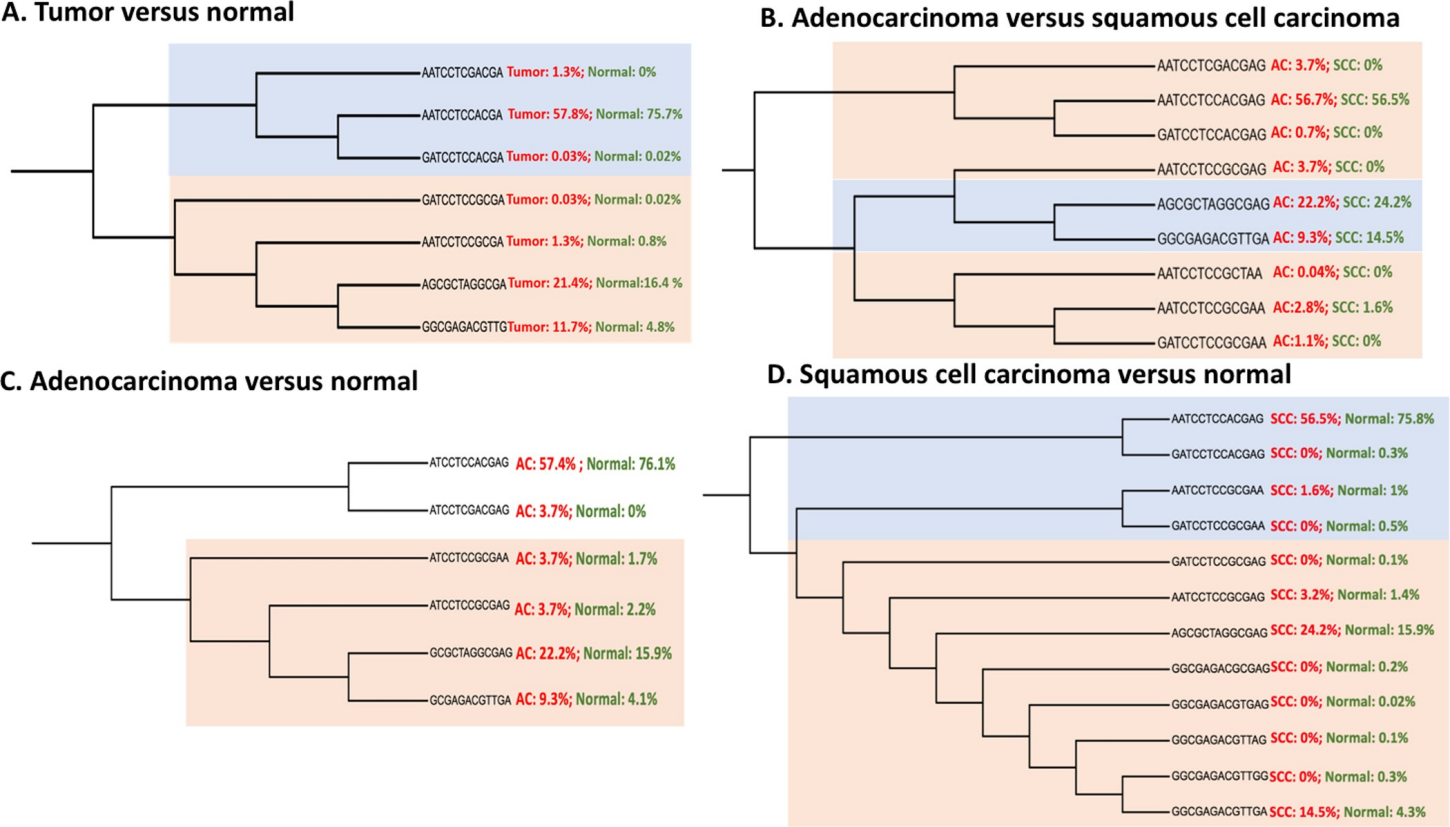
Haplotype trees by subtypes of lung cancer. A. SNPs that defined the LD block among tumor and normal lung samples were used to reconstruct haplotypes using PHASE version 2.1.1. The haplotypes were analyzed based on molecular diversity indices. The minimum spanning tree among haplotypes were computed based on pairwise differences in Arlequin version 3.5. The first cluster of haplotypes (in gray shade) were more likely to present in normal lung tissue samples compared to tumor tissue samples, especially for “AATCCTCCACGA”. The second cluster of haplotypes (in pink shade) were more likely to show in tumor tissue samples compared to normal samples, especially for the last three haplotypes: “AATCCTCCGCGA”, “AGCGCTAGGCGA”, and “GGCGAGACGTTG”. B. SNPs that defined the LD block among AC and SCC lung samples were used to reconstruct haplotypes in PHASE version 2.1.1. The minimum spanning tree among haplotypes were computed based on pairwise differences in Arlequin version 3.5. The clusters in pink shade show haplotypes that were more likely to present in AC compared to SCC tissues. The cluster in grey shade show haplotypes that were more likely to present in SCC samples compared to AC tissue samples. The haplotypes “AGCGCTAGGCGAG” and “GGCGAGACGTTGA” were more likely to appear in SCC compared to AC tissue samples. C. SNPs that defined the LD block among AC and normal lung samples were used to reconstruct haplotypes in PHASE version 2.1.1. The minimum spanning tree among haplotypes were computed based on pairwise differences in Arlequin version 3.5. The second cluster of haplotypes (in pink shade) were more likely to show in AC tissue samples compared to normal samples, especially for haplotypes: “ATCCTCCGCGAA”, “ATCCTCCGCGAG”, “GCGCTAGGCGAG”, and “GCGAGACGTTGA”. D. SNPs that defined the LD block among SCC and normal lung samples were used to reconstruct haplotypes in PHASE. The minimum spanning tree among haplotypes were computed based on pairwise differences in Arlequin. The first cluster of haplotypes (in grey shade) were more likely to present in normal lung tissue samples compared to SCC samples, especially for “AATCCTCCACGAG”. The second cluster of haplotypes (in pink shade) were more likely to show in SCC tissue samples compared to normal samples, especially for the haplotypes: “AATCCTCCGCGAG”, “AGCGCTAGGCGAG”, and “GGCGAGACGTTGA”.

Two main clusters of haplotypes were shown when comparing lung tumor tissue samples with normal lung tissue samples. Haplotype “AATCCTCCACGA” in the first cluster (in gray) was more likely to present in normal tissue samples. Haplotypes “AATCCTCCGCGA”, “AGCGCTAGGCGA”, and “GGCGAGACGTTG” in the second cluster (in pink) were more likely to present in lung tumor tissue samples (Fig 3A). AC lung tissue samples had a lower probability of showing haplotype “AGCGCTAGGCGAG” and “GGCGAGACGTTGA” in the second cluster (in blue) compared to SCC lung tissue samples (Fig 3B). When comparing AC lung tissue samples with normal lung tissue samples, the haplotypes in the second cluster were more likely to present in AC tissue samples compared to normal lung tissue samples, including “ATCCTCCGCGAA”, “ATCCTCCGCGAG”, “GCGCTAGGCGAG”, and “GCGAGACGTTGA” (Fig 3C). For the comparison between SCC tissue samples and normal lung tissue samples, haplotypes in the second main cluster are more likely to present in SCC tissues compared to normal tissues, including “AATCCTCCGCGAA”, “AATCCTCCGCGAG”, “AGCGTAGGCGAG”, and “GGCGAGACGTTGA” (Fig 3D).

### 3.6 mRNA expression analysis and validation using publicly available microarray dataset

**Fig 4:**
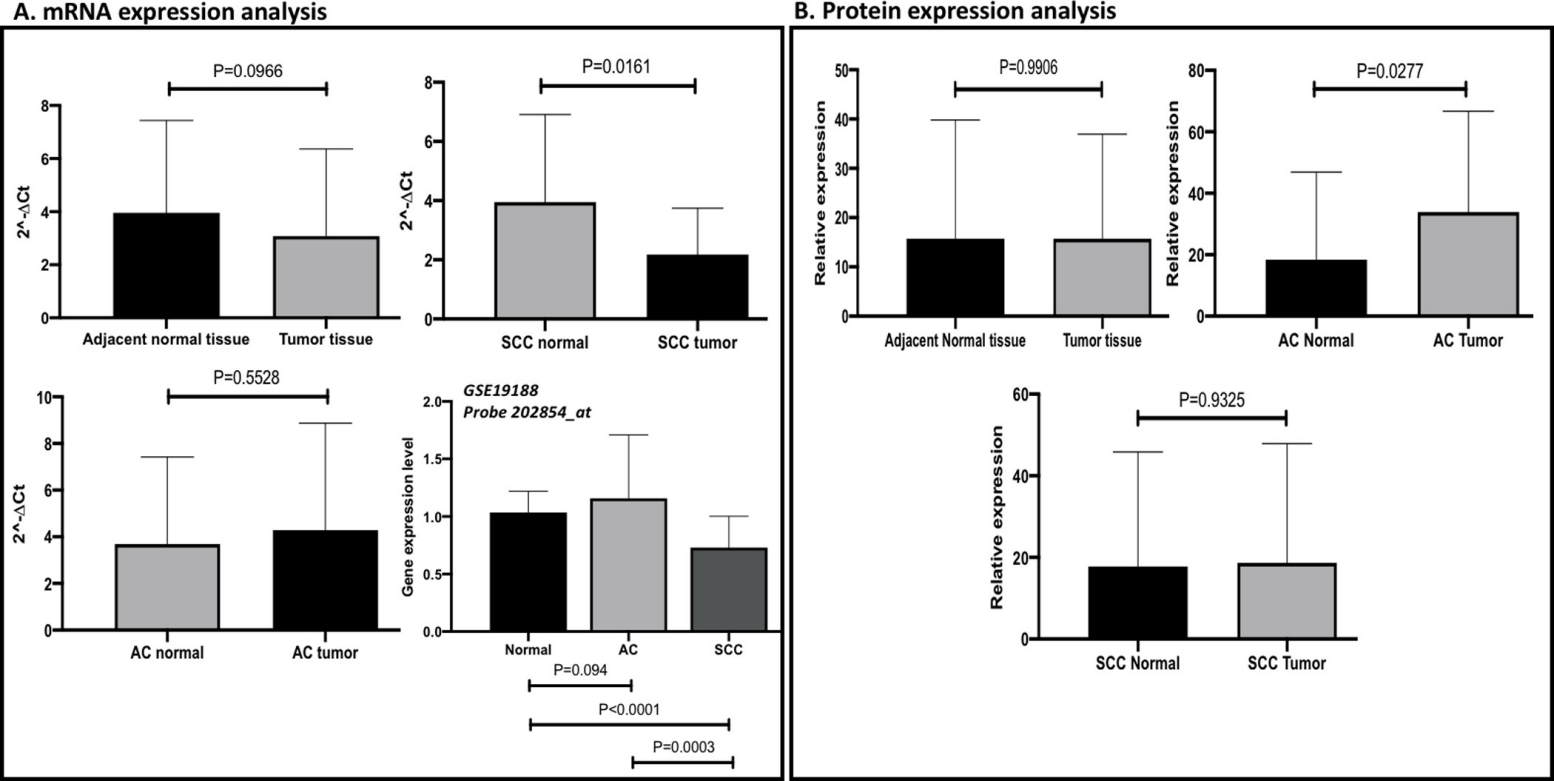
mRNA and protein expression analyses. A. Paired t-test (N = 62 pairs) was conducted for fresh frozen lung tissue samples. One-way ANOVA were performed for GEO dataset (GSE 19188). The normalized values for normal lung tissues were compared to normalized values of tumor tissues. *PARP1* was relatively underexpressed among tumor tissues (especially SCC tissues, p = 0.016) compared to adjacent normal tissues. *PARP1* was slightly overexpressed in AC tumor issues compared to paired AC normal tissues. The result of GEO microarray dataset showed that *PARP1* was relatively overexpressed in AC lung tissue samples (p<0.0001) and underexpressed in SCC tissues (p = 0.0003) compared to normal lung tissues. The results of GEO were consistent with our results. The HPRT probe used in the GEO dataset was 202854_at. B. A subset of the paired samples (N = 29 pairs) were included for protein expression analysis. The normalized values for paired normal lung tissues were compared to normalized values for AC tissues and for SCC tissues using a paired t-test. The binding site of antibody we used covers part of PARP catalytic domain and whole PARP alpha-helical domain. The protein expression of *PARP1* was similar between lung tumor tissues and paired normal lung tissue samples (p = 0.991). *PARP1* was significantly overexpressed in AC tissues compared to paired normal tissue samples (p = 0.028). No significant difference of *PARP1* protein expression was observed between SCC and normal lung tissues (p = 0.933).

*PARP1* was downregulated among lung tumor tissue samples compared to paired normal lung tissue samples. The stratified analysis showed that the *PARP1* expression was slightly higher among AC tissue samples compared to paired normal lung tissue samples, while *PARP1* was downregulated significantly in SCC lung tissue samples compared to paired normal lung tissue samples (Fig 4A). The unpaired analysis compared the means of two independent groups and included all paired and unpaired samples. The unpaired analysis showed similar results with paired analysis, which suggested fewer confounding issues. The unpaired mRNA expression result is shown in the S7 Fig in S1 File.

To validate the results, we used the GEO microarray dataset, which was deposited by Hou et al. research team. They obtained 156 fresh frozen lung tissue samples, including 91 tumor tissues and 65 adjacent normal lung tissues, from 91 patients who received curative surgical resection from 1992 to 1998, or from 1996 to 2004 at the center. From the total RNA, mRNA was processed on Affymetrix U133 Plus 2.0 arrays following standard protocols. Most patients had early-stage lung cancer. More than 90% of patients were of European ancestry. The demographic background of the publicly available dataset is comparable to our study population. *PARP1* probe used in this analysis was 208644_at, which included the epitope used in protein expression analysis. The results of the GEO dataset showed that *PARP1* was relatively overexpressed in AC lung tissue samples but was significantly underexpressed in SCC lung tissue samples compared to normal lung tissue samples, which is consistent with our results (Fig 4A).

### 3.7 Protein expression analysis

The *PARP1* antibody (F-2) was raised against the amino acids 764–1014 of PARP of human origin. The binding site of antibody covers part of PARP catalytic domain and whole PARP alpha-helical domain. The results showed that the expression of *PARP1* was similar between lung tumor tissue samples compared to paired normal lung tissue samples. *PARP1* was significantly overexpressed in AC tissue samples compared to paired normal lung tissue samples, but there was no significant difference when comparing SCC lung tissue samples to paired normal lung tissue samples (Fig 4B).

## 4. Discussion

To further explore the role of the *PARP1* gene in NSCLC, our study found that the association between *PARP1* and NSCLC varies by NSCLC subtypes, allelic heterogeneity, and specific haplotypes in patients from our study population. Our study is the first study to use both genotyped and imputed SNP data to analyze the association between *PARP1* alleles/haplotype and NSCLC subtypes. Interaction analysis of SNPs in *PARP1* showed that the high-risk genotype combination derived from rs3219073, rs1805415, and rs1805414 contributed increasing risk of AC, SCC and lung tumor phenotypes. Further, specific clusters of haplotypes identified was able to distinguish the NSCLC tumor tissue subtypes and normal tissues. The presence of these alleles and haplotypes further correlated with mRNA and protein expression in NSCLC tissues.

While our study reports that rs1136410 is associated with increased risk of NSCLC, some studies have reported that rs1136410 is associated with decreased risk of lung cancer [42] or a better survival outcome [43]. The conflicting results is likely due to the genetic heterogeneity contributed by ethnic or genetic background of the population being studied and whether the SNP was correlated with lung cancer at the subtype level. Another study by Wang HT et al. reported that allele G on *PARP1*-rs3219073 may reduce the risk of lung cancer among Asians [21]. In our population with a majority of European ancestry and specifically in NSCLC, we found *PARP1*-rs3219073 alone may not be associated with lung cancer, but rs3219073, rs1805415, and rs1805414 together could differentiate the lung tumor and normal tissues in MDR analysis (Table 2).

To comprehensively investigate the role of *PARP1* in lung cancer, our study explored the association between *PARP1* and lung cancer based on allelic/genotypic association, SNP-SNP interaction, and haplotype analysis. We found one SNP or allele may not fully explain the role of *PARP1* in lung cancer. The alleles of two SNPs (rs1805414-G, rs1805404-A) were significantly associated with both lung tumor and SCC tissue compared to normal lung tissues. SNP (rs1136410-G) was associated with lung tumor tissues compared to normal with a P-value of 0.062 (OR = 2.218; 95%CI:0.944, 5.212). However, those SNPs could not differentiate between AC and normal tissues or between AC and SCC tissues at allelic/genotypic level.

Increasingly studies have shown that SNPs are potentially strong candidate biomarkers for identifying susceptibility to lung cancer. We believe this is the first study that evaluated SNP-SNP interaction in *PARP1* and showed a significant interaction between rs3219073, rs1805414, and rs1805415 that contributed to an increased risk of AC and SCC. The strategic selection of these genotyped SNPs based on functional importance alongside imputed SNPs that share an LD block has the potential to identify complex biological links among SNPs derived from *PARP1* functional domains and cancer processes. For example, rs3219073 is located in the DNA-damage checkpoint and implicated in DNA-repair pathways while rs1805414, and rs1805415 are in the DNA binding domain [44, 45]. Therefore, the combined epistatic effect of the three SNPs may be important in explaining severity of NSCLC or development of AC vs SCC. Validation of our results in an independent data set will provide further insight into the role of these SNPs in NSCLC etiology.

Studies have shown that *PARP1* accelerates the recruitment of DNA repair proteins to strand interruption through interacting with the scaffold protein, such as XRCC1 [44, 45]. Particularly SNP rs1136410 on both PARP catalytic and PARP alpha-helical domains [46, 47] have been linked to the substitution of alanine for valine which affects the base excision repair capacity [48]. Another study showed that rs1136410 (i.e. Val762Ala polymorphism) reduces the enzymatic activity of *PARP1* through increasing Km, suggesting a possible cancer risk of carriers of rs1136410 [49]. Our study is consistent with most studies [50–52], and we also report rs1136410 is associated with increased risk of NSCLC.

A recent study in an Asian population constructed haplotypes using SNPs from *PARP1* coding regions [Asp81Asp, C801T in exon 2; Alal88Thr, G721A in exon 4; Ala284Ala, C1011T in exon 7; His613Glu, C1998G in exon 13; Val762Ala, T2444C in exon 17; and Cys908Thr, G2882A in exon 20], and tested for association with lung cancer [22]. These haplotypes were not significantly associated with lung cancer [22]. In our study which were mainly of European ancestry, haplotype analyses showed that the SNPs rs1136410 (Val762Ala) and rs1805414 (Ala284Ala) along with other genotyped or imputed SNPs consist of haplotypes (“AATCCTCCGCGA”, “AGCGCTAGGCGA”, “GGCGAGACGTTG”) that were more likely to present in lung tumor tissues compared to normal tissues (Fig 3A). This unique cluster of haplotypes (Fig 3A) further illustrates the importance of specific alleles, that define the haplotype which encompasses functional domains important in the processes of NSCLC. The “G” allele of rs1805414 in the DNA-binding domain only showed in the haplotypes cluster with a higher proportion of tumor samples, while “A” allele of rs1805414 showed in the haplotypes cluster with a higher proportion of normal samples. Interestingly the DNA binding domain is an important target for the *PARP1* inhibitor antitumor drug design [53]. Further, specific haplotypes were more likely to distinguish AC from SCC phenotypes.

Two studies have shown that *PARP1* is highly expressed in small-cell lung cancer cell lines at the mRNA and protein levels in the USA, and SCLC cell lines was significantly more sensitive to PARP inhibitors than were NSCLC cell lines [44, 54]. Another study showed that *PARP1* is associated with longer progression-free survival in limited-stage small cell lung cancer among South Korean population [55]. In our study, the mRNA expression analyses showed that *PARP1* was upregulated in AC lung tissue samples but downregulated in SCC tissue samples compared to paired normal lung tissue samples (Fig 4A). The protein expression results showed the similar result that *PARP1* was significantly upregulated in AC tissue samples compared to paired normal lung tissue samples (Fig 4B). The role of *PARP1* in lung AC is consistent with a previous study reporting that *PARP1* enhances lung AC metastasis [15]. Our study further explored that specific alleles/SNPs, interaction between functional SNPs and haplotypes (containing selected functional SNPs) on *PARP1* may contribute to the increased risk of lung AC. However, the effect of *PARP1* on lung SCC should be further investigated.

In conclusion, our study found that *PARP1* haplotypes can better predict NSCLC subtypes. The interaction between SNPs in *PARP1*confers increased risk of developing AC and SCC in the population of European ancestry. Our study suggests that the combination of functional genotyped SNPs and imputed SNPs that define haplotypes of *PARP1* may serve as a better predictor in NSCLC development and prognosis compared to single alleles. More studies should be conducted to further validate the role of *PARP1* in various ethnic groups with different stages and subtypes of lung cancer.

## Supporting information

S1 File (PPTX)

S2 File (DOCX)

S3 File (ZIP)
