# Supplementary material for: Association of PARP1-specific polymorphisms and haplotypes with non-small cell lung cancer subtypes
Source: PLoS One. 2020 Dec 7;15(12):e0243509. doi: 10.1371/journal.pone.0243509 (PMC7721167; doi:10.1371/journal.pone.0243509)
Supplement: S1 File — (PPTX) [file pone.0243509.s001.pptx]

## Slide 1
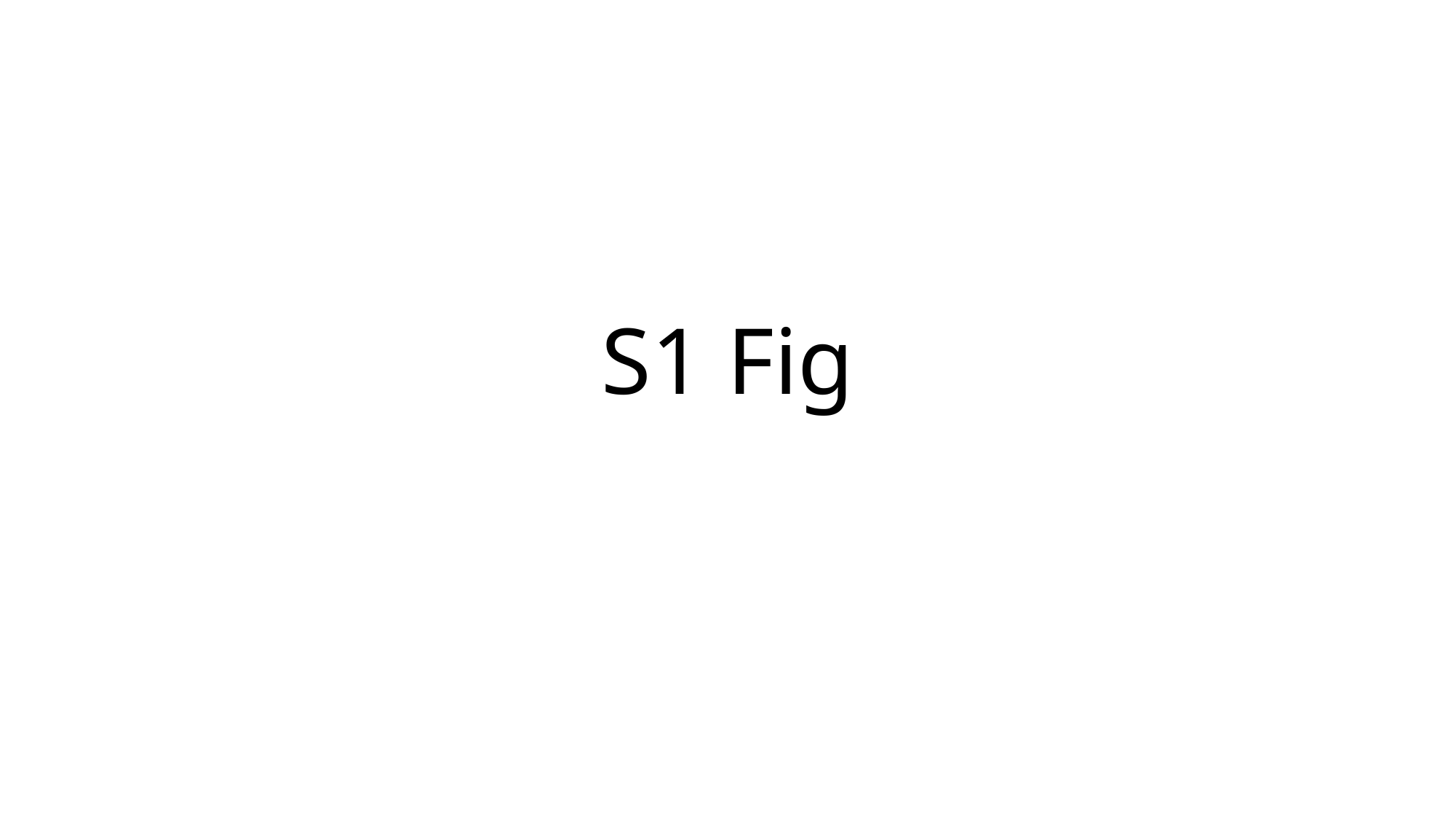

# S1 Fig

## Slide 2
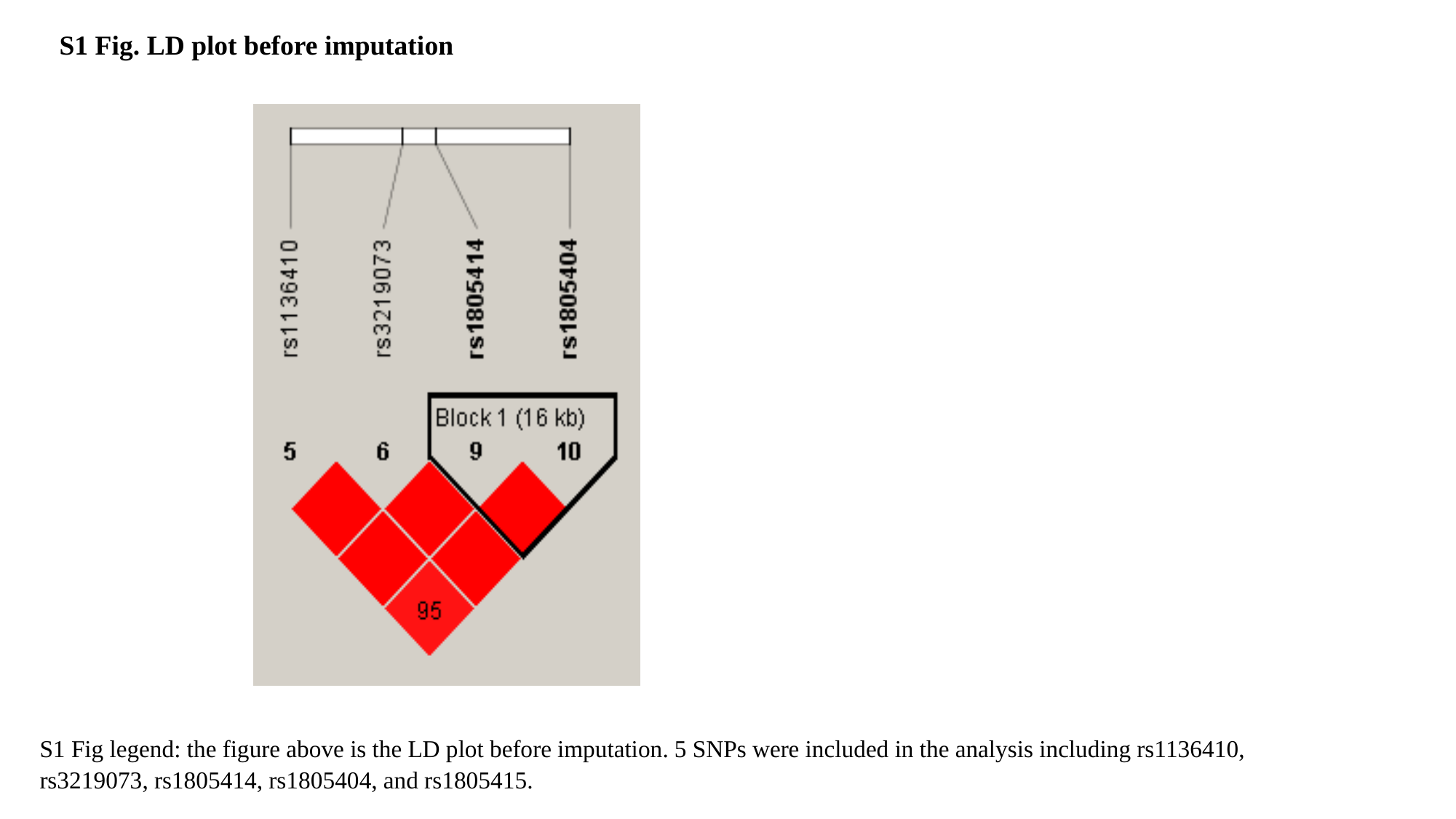

S1 Fig. LD plot before imputation
S1 Fig legend: the figure above is the LD plot before imputation. 5 SNPs were included in the analysis including rs1136410, rs3219073, rs1805414, rs1805404, and rs1805415.

## Slide 3
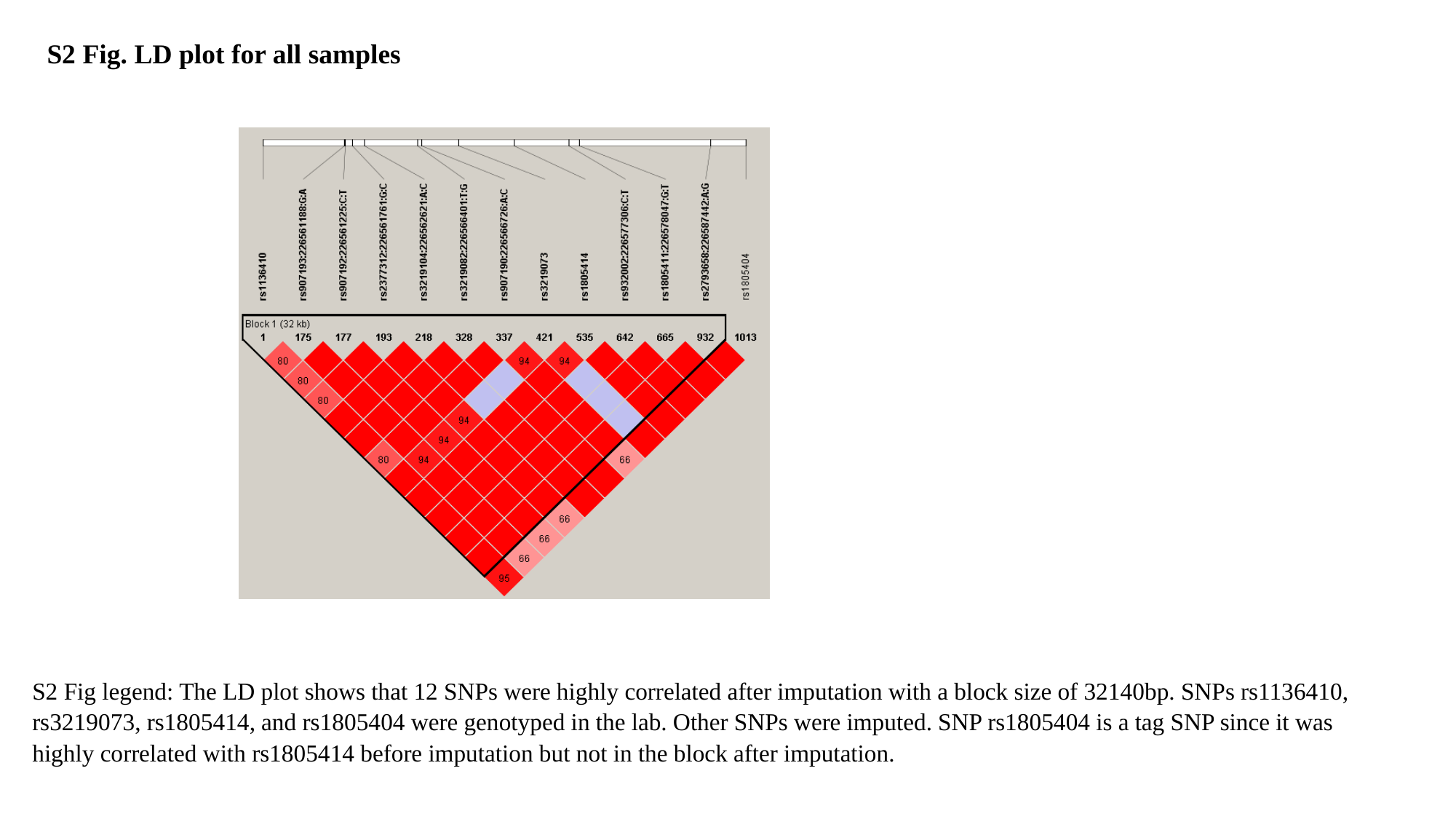

S2 Fig. LD plot for all samples
S2 Fig legend: The LD plot shows that 12 SNPs were highly correlated after imputation with a block size of 32140bp. SNPs rs1136410, rs3219073, rs1805414, and rs1805404 were genotyped in the lab. Other SNPs were imputed. SNP rs1805404 is a tag SNP since it was highly correlated with rs1805414 before imputation but not in the block after imputation.

## Slide 4
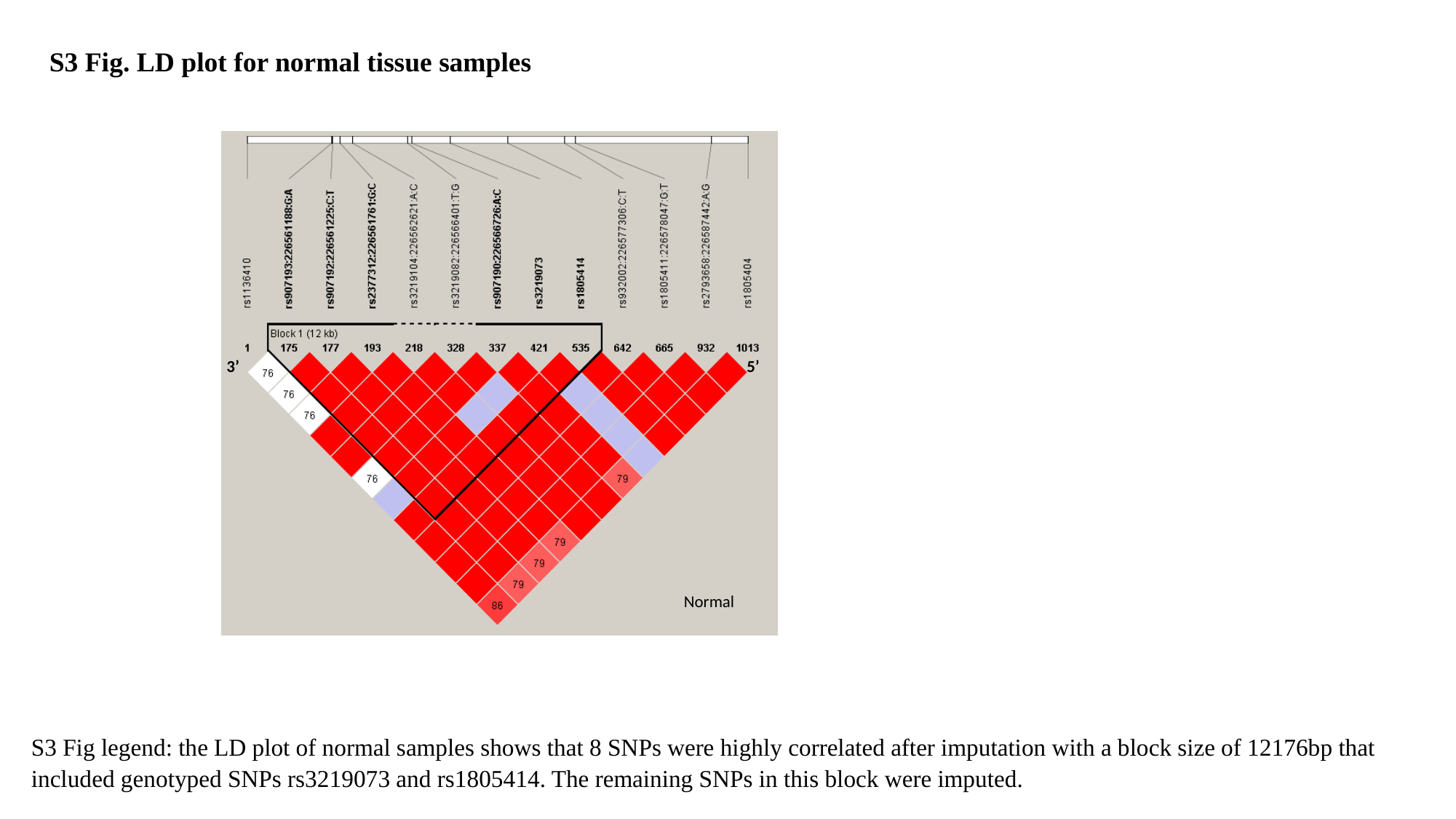

S3 Fig. LD plot for normal tissue samples
3’
5’
Normal
S3 Fig legend: the LD plot of normal samples shows that 8 SNPs were highly correlated after imputation with a block size of 12176bp that included genotyped SNPs rs3219073 and rs1805414. The remaining SNPs in this block were imputed.

## Slide 5
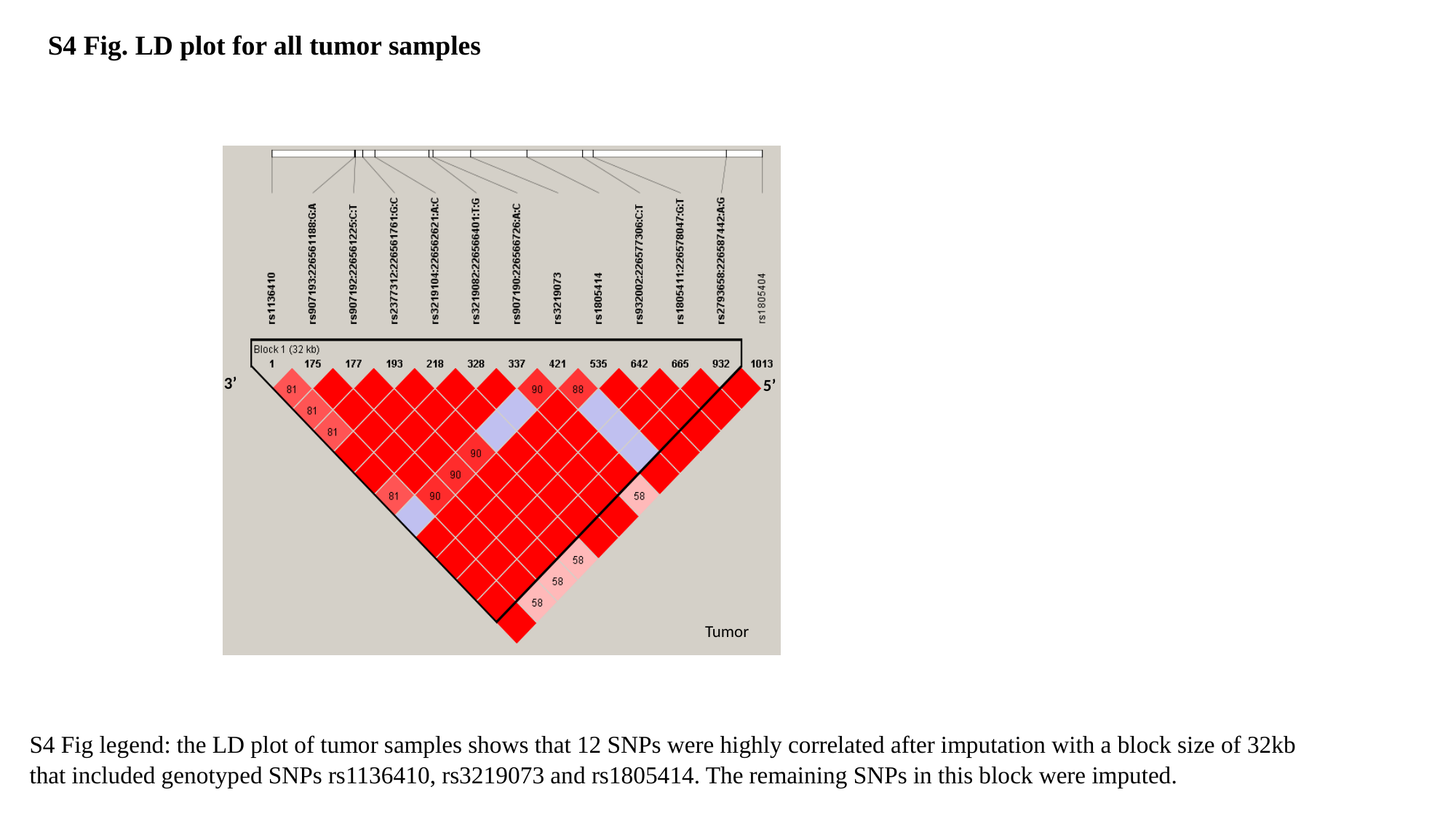

S4 Fig. LD plot for all tumor samples
3’
5’
Tumor
S4 Fig legend: the LD plot of tumor samples shows that 12 SNPs were highly correlated after imputation with a block size of 32kb that included genotyped SNPs rs1136410, rs3219073 and rs1805414. The remaining SNPs in this block were imputed.

## Slide 6
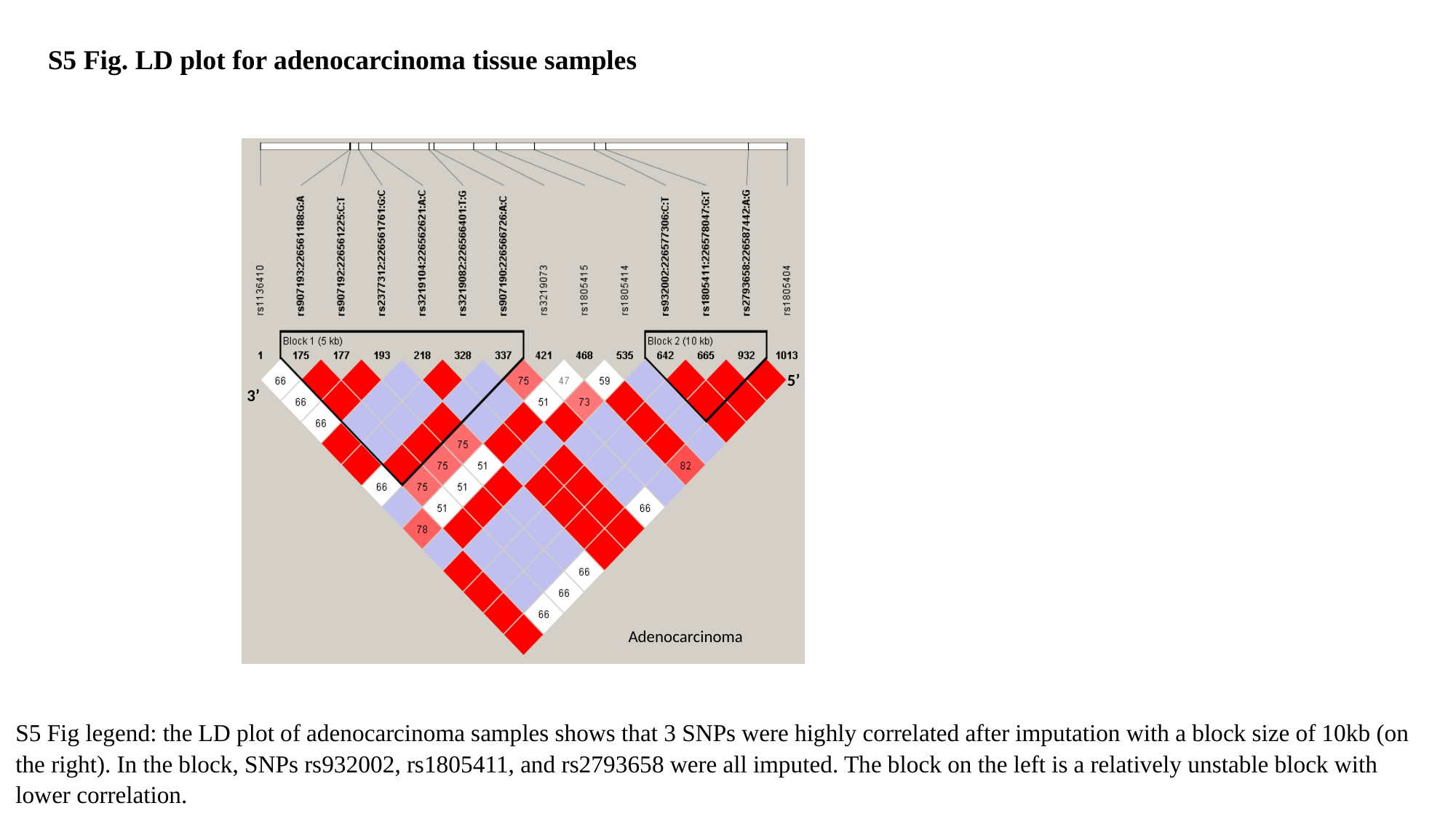

S5 Fig. LD plot for adenocarcinoma tissue samples
5’
3’
Adenocarcinoma
S5 Fig legend: the LD plot of adenocarcinoma samples shows that 3 SNPs were highly correlated after imputation with a block size of 10kb (on the right). In the block, SNPs rs932002, rs1805411, and rs2793658 were all imputed. The block on the left is a relatively unstable block with lower correlation.

## Slide 7
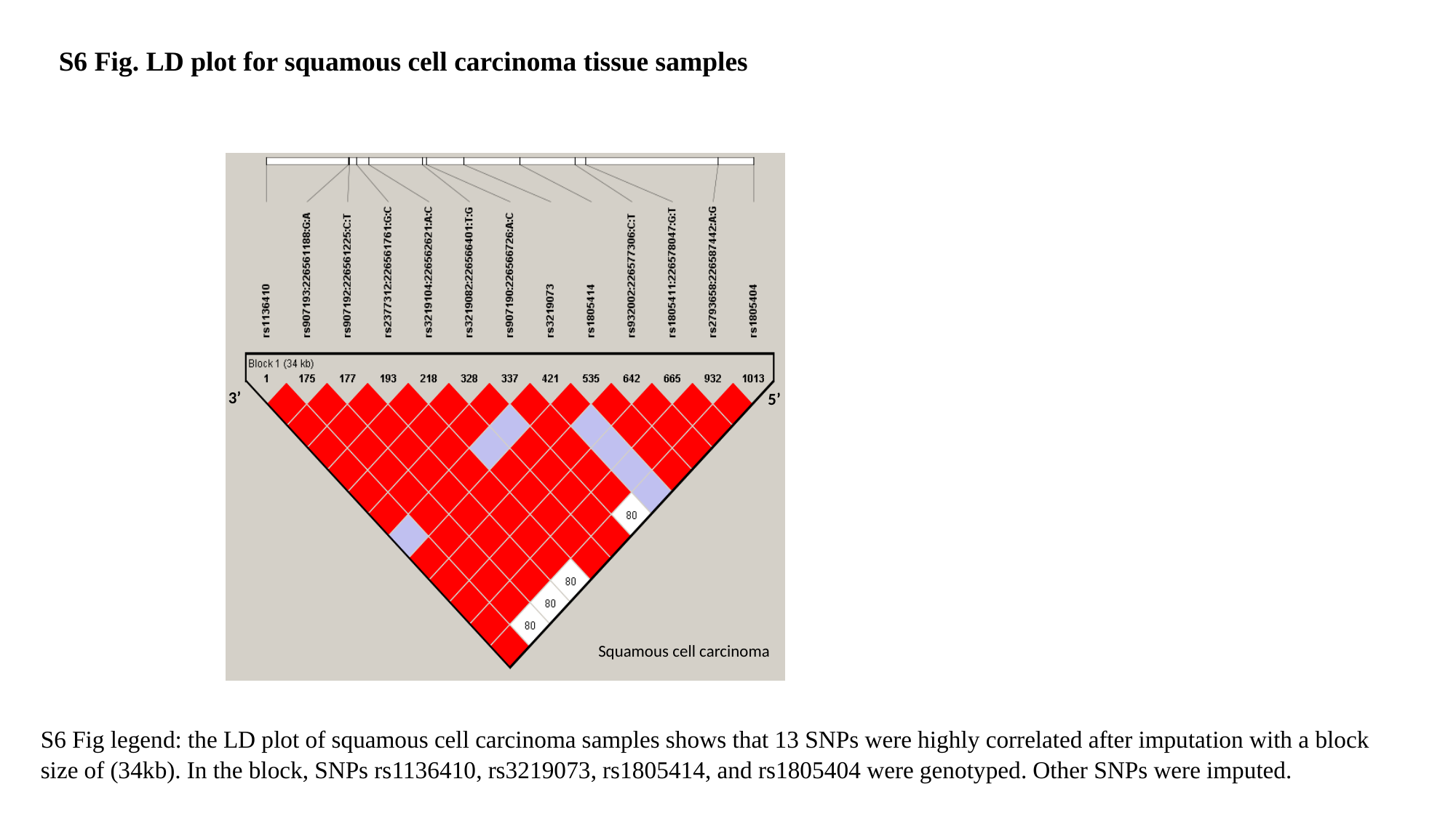

S6 Fig. LD plot for squamous cell carcinoma tissue samples
3’
5’
Squamous cell carcinoma
S6 Fig legend: the LD plot of squamous cell carcinoma samples shows that 13 SNPs were highly correlated after imputation with a block size of (34kb). In the block, SNPs rs1136410, rs3219073, rs1805414, and rs1805404 were genotyped. Other SNPs were imputed.

## Slide 8
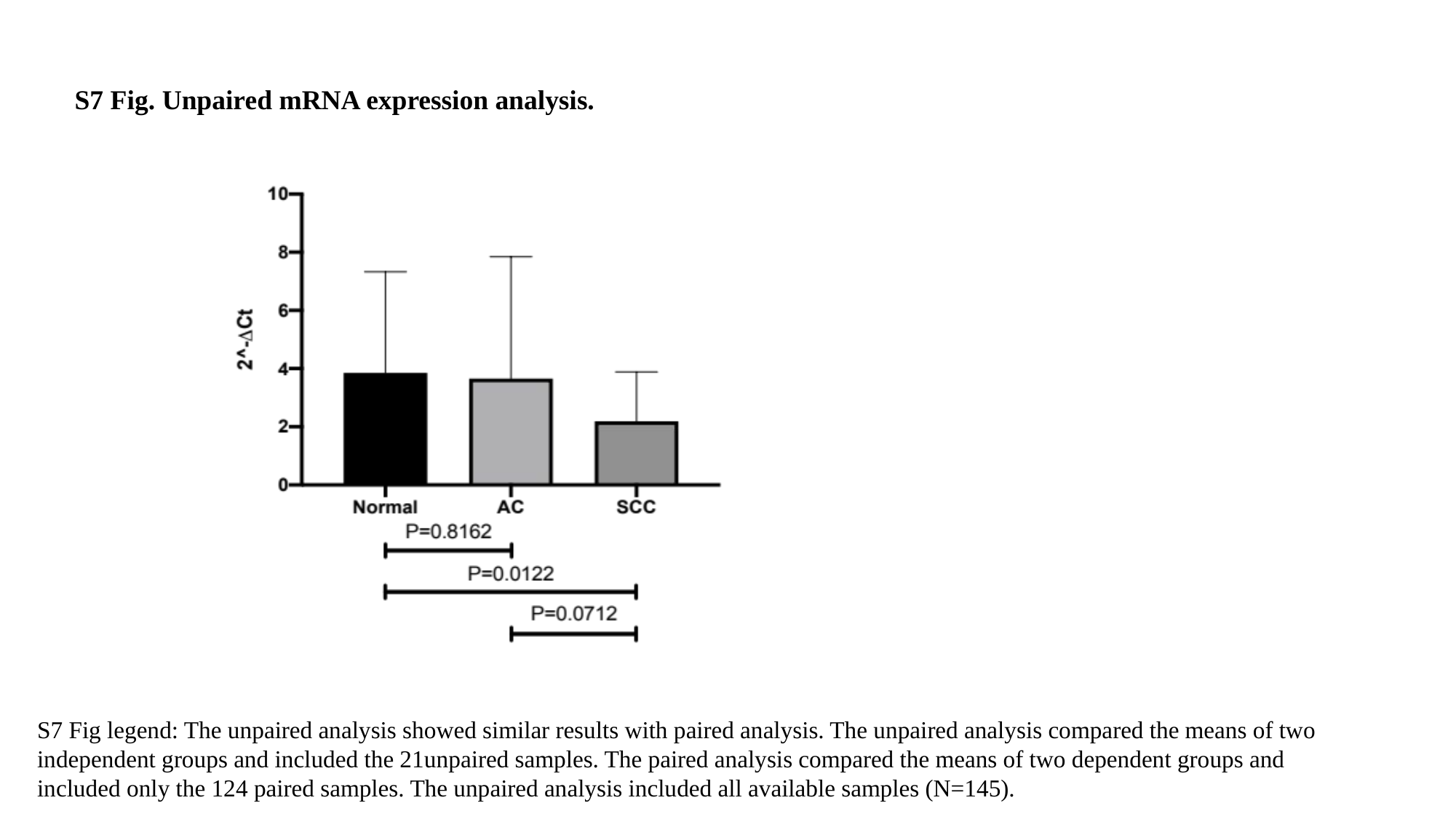

S7 Fig. Unpaired mRNA expression analysis.
S7 Fig legend: The unpaired analysis showed similar results with paired analysis. The unpaired analysis compared the means of two independent groups and included the 21unpaired samples. The paired analysis compared the means of two dependent groups and included only the 124 paired samples. The unpaired analysis included all available samples (N=145).
