# Supplementary material for: Association of PARP1-specific polymorphisms and haplotypes with non-small cell lung cancer subtypes
Source: PLoS One. 2020 Dec 7;15(12):e0243509. doi: 10.1371/journal.pone.0243509 (PMC7721167; doi:10.1371/journal.pone.0243509)
Supplement: S2 File — (DOCX) [file pone.0243509.s002.docx]

**S1 Table. GEO dataset selection criteria**

| **Inclusion criteria** | **Exclusion criteria** |
| --- | --- |
| - Lung cancer samples - Non-small cell lung cancer samples - Majority samples are early stage lung cancer - Caucasians | - No comparison group (normal lung samples) - Not enough sample size |

**S2 Table. Demographic background of study population**

|  | **AC tissues** | **SCC tissues** | **Other types** | **Normal tissues** | **Total tissue number** |
| --- | --- | --- | --- | --- | --- |
| **Paired samples**  **(N, pairs)** | 22 | 24 | 16 | N/A | 62 |
| **Unpaired samples (N)** | 8 | 8 | 4 | 1 | 21 |
| **Total tissue number (N)** | 52 | 56 | 36 | 1 | 145 |

**S3 Table. Minor allele frequency of genotyped SNPs in study population**

| **SNPs** | **Position** | **MAF** |
| --- | --- | --- |
| **rs1136410** | **226555302** | **G: 9.09%** |
| **rs3219073** | **226569375** | **C: 20.36%** |
| **rs1805415** | **226570840** | **T: 14.09%** |
| **rs1805414** | **226573364** | **G: 32.99%** |
| **rs1805404** | **226589958** | **A: 10.56%** |
